# Experimental arthritis and *Porphyromonas gingivalis* administration synergistically decrease bone regeneration in femoral cortical defects

**DOI:** 10.1038/s41598-019-56265-6

**Published:** 2019-12-27

**Authors:** Go Okumura, Naoki Kondo, Keisuke Sato, Kazuhisa Yamazaki, Hayato Ohshima, Hiroyuki Kawashima, Akira Ogose, Naoto Endo

**Affiliations:** 1 Division of Orthopedic Surgery, Department of Regenerative and Transplant Medicine, Niigata University Graduate School of Medical and Dental Sciences, Niigata, Japan; 2 Division of Periodontology, Department of Oral Science, Niigata University Graduate School of Medical and Dental Sciences, Niigata, Japan; 3 Research Unit for Oral-Systemic Connection, Division of Oral Science for Health Promotion, Niigata University Graduate School of Medical and Dental Sciences, Niigata, Japan; 4 Division of Anatomy and Cell Biology of the Hard Tissue, Department of Tissue Regeneration and Reconstruction, Niigata University Graduate School of Medical and Dental Sciences, Niigata, Japan

**Keywords:** Bone, Periodontitis, Rheumatoid arthritis, Osteoimmunology

## Abstract

*Porphyromonas gingivalis* infection can lead to periodontitis and dysbiosis, which are known risk factors for rheumatoid arthritis (RA). We investigated whether *P. gingivalis* administration affected bone regeneration in mice with or without arthritis. We administered *P. gingivalis* to male DBA/1 J mice that were or were not sensitised to type II collagen-induced arthritis (CIA). All mice underwent drilling of bilateral femurs. We histologically evaluated new bone regeneration (bone volume of the defect [BVd]/tissue volume of the defect [TVd]) using micro-computed tomography (micro-CT), osteoclast number/bone area, and active osteoblast surface/bone surface (Ob.S/BS). We measured serum cytokine levels and bone mineral density of the proximal tibia using micro-CT. CIA resulted in significantly reduced bone regeneration (BVd/TVd) at all time-points, whereas *P. gingivalis* administration showed similar effects at 2 weeks postoperatively. CIA resulted in higher osteoclast number/bone area and lower Ob.S/BS at 2 and 3 weeks postoperatively, respectively. However, *P. gingivalis* administration resulted in lower Ob.S/BS only at 2 weeks postoperatively. During later-stage bone regeneration, CIA and *P. gingivalis* administration synergistically decreased BVd/TVd, increased serum tumour necrosis factor-α, and resulted in the lowest bone mineral density. Therefore, RA and dysbiosis could be risk factors for prolonged fracture healing.

## Introduction

In rheumatoid arthritis (RA), increased synovitis-related inflammatory cytokines induce osteoclast differentiation and cause secondary osteoporosis^1,2^. Therefore, patients with RA are at higher risk for fractures than healthy individuals^3–6^. Furthermore, delays in fracture healing have been observed in animal models of RA, diabetes, and sepsis^7–9^, indicating that systemic inflammation has a negative impact on bone regeneration. Bone regeneration is a complex and highly regulated process, with consecutive and overlapping phases of inflammation, granulation tissue formation, intramembranous and endochondral ossification, and remodelling. Recent reports have shown an association between the bone regeneration process and immune cells^10^. Systemic inflammatory states such as RA can alter immune responses, thereby prolonging fracture healing and increasing the risk of non-union via mechanisms that are not well understood^11^. Inflammatory cytokines such as tumour necrosis factor (TNF)-α and interleukin-6 (IL-6) may be involved in the process^12^, as TNF-α and IL-6 play crucial regulatory functions in all stages of bone repair^13–17^. Moreover, the pathology of RA is closely related to these pro-inflammatory cytokines^18^.

Bone destruction can result from T_H_17 cells that emerge in RA and periodontal disease^19^. Periodontal diseases are common and involve exacerbation of inflammation due to periodontal tissue infection with pathogens like *Porphyromonas gingivalis*, that induce excessive osteoclast differentiation and lead to destruction of the alveolar bone. There is a close relationship between RA and periodontal diseases, which are often complicated by RA^20–22^. In addition, RA disease activity may improve with treatment commonly used for periodontal diseases^23,24^. It is believed that infection with periodontal pathogens is associated with the production of anti-cyclic citrullinated peptide antibodies^25^, the induction of dysbiosis by *P. gingivalis*^26,27^ leads to immune dysfunction, which induces autoimmune disorders^28^. Several studies have reported that periodontal diseases (or the administration of periodontal pathogens) exacerbate arthritis in experimental models^29–31^. Sato *et al*. have also reported that oral administration of *P. gingivalis* (dysbiosis model) caused changes in the intestinal microflora and immunity, and exacerbated arthritis^32^.

In cases where the administration of *P. gingivalis* exacerbates systemic inflammation to the extent of deteriorating arthritis, it may also be expected to have a negative impact on fracture healing. However, no basic studies have examined the effects of *P. gingivalis* administration and collagen-induced arthritis (CIA) on bone regeneration of the limbs. In this study, we induced cortical bone damage in the femurs of mice and analysed the impact of CIA and *P. gingivalis* administration on bone restoration. We hypothesised that bone regeneration is suppressed in CIA mice, and administration of *P. gingivalis* leads to an interaction, that synergistically suppresses bone regeneration. Validation of this hypothesis may indicate that treatment used for periodontal diseases and dysbiosis may also be used to treat fractures in RA patients.

## Results

### *P. gingivalis* administration exacerbated the arthritis score

**Figure 1:**
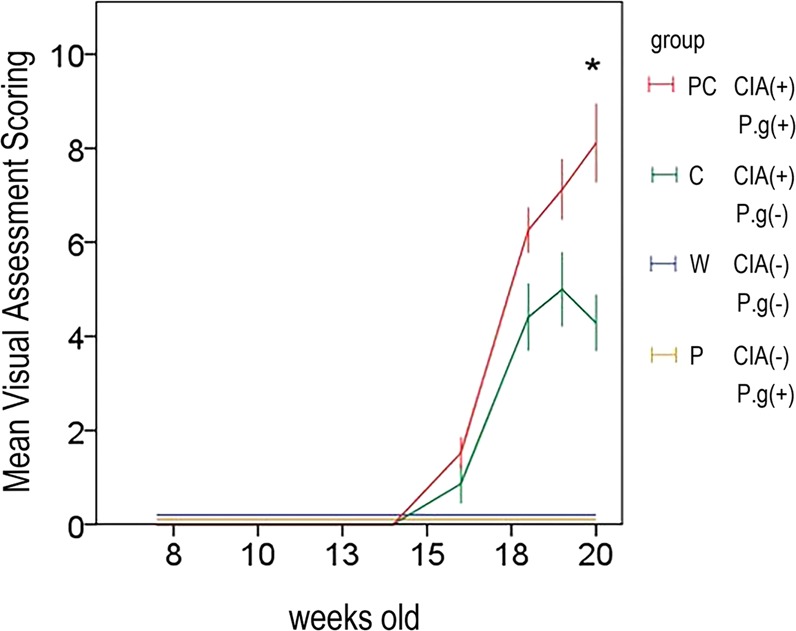
Visual assessment scores (VAS) are presented as mean ± standard error of the mean (SEM). VAS of 20-week-old mice in group PC were significantly higher than those of group P. All scores in groups W and P were zero. *P < 0.01 versus group C. Group W, non-arthritic mice without bacterial administration. Group C, arthritic mice without bacterial administration. Group P, non-arthritic mice with bacterial administration. Group PC, arthritic mice with bacterial administration.

No arthritis was observed in the control (group W) or *P. gingivalis* administration without CIA (group P) group. At 18, 19, and 20 weeks of age, the arthritis scores of the CIA without *P. gingivalis* administration (group C) group were 4.5 ± 0.7, 5.1 ± 0.7, and 4.6 ± 0.6, while those of the CIA with *P. gingivalis* administration group (group PC) were 6.2 ± 0.5, 7.1 ± 0.7, and 8.3 ± 0.9, respectively. Group PC had significantly higher scores at an age of 20 weeks (p = 0.054, p = 0.060, and p = 0.006 at 18, 19, and 20 weeks, respectively) (Fig. 1).

### CIA and *P. gingivalis* administration decreased ‘bone:tissue volume of the defect’ during bone regeneration in cortical defects and showed synergistic reductions in ‘bone:tissue volume of the defect’ at 4 weeks postoperatively

Significantly reduced bone regeneration (bone:tissue volume of the defect [BVd/TVd]) occurred with CIA over time, whereas *P. gingivalis* administration resulted in similar effects only at 2 weeks postoperatively. CIA and *P. gingivalis* administration resulted in a synergistic reduction in bone regeneration during the later stages of bone regeneration. Group PC had the lowest BVd/TVd at 4 weeks postoperatively.

**Figure 2:**
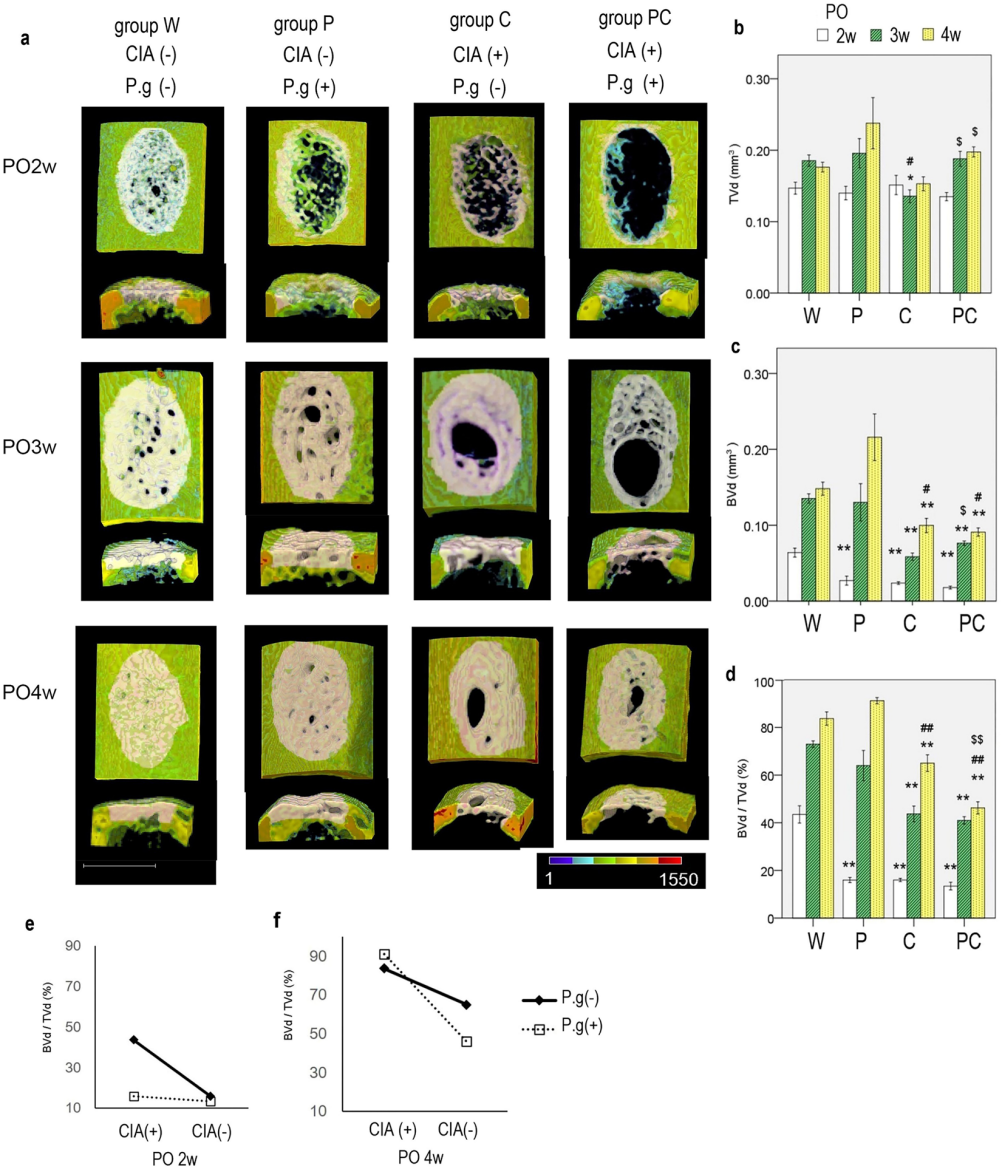
(**a**) Three-dimensional computed tomography images of the uni-cortical defect of the left femur are shown (upper, ventral surface of femur; lower, axial section of the area). At 4 weeks postoperatively (PO4w), groups W and P showed higher bone regeneration, with complete full thickness bridging, similar to non-injured cortical bone; in contrast, groups C and PC showed lower bone regeneration. Pink area represents the analysed volume of interest in the well-mineralised regenerated bone area. n = 5–8 per group. *White bar* represents 600 μm. Coloured area represents bone mineral density in accordance with the colour scale bar (mg/cm^3^). (**b**) Quantitative evaluation of the tissue volume in the defect lesion (TVd), (**c**) well-mineralised bone volume of the defect lesion (BVd), and (**d**) bone volume fraction (BVd/TVd) of the defect lesion are shown. Data are presented as mean ± SEM. n = 5–8 per group. White, black shaded, and dot shaded columns represent 2, 3, and 4 weeks postoperatively, respectively. *P < 0.05, **P < 0.01 versus group W at the same time point. ^#^P < 0.05, ^##^P < 0.01 versus group P at the same time point. ^$^P < 0.05, ^$$^P < 0.01 versus group C at the same time point. (**e**,**f**) Interaction of collagen-induced arthritis (CIA) and *Porphyromonas gingivalis* administration (P.g) with respect to mean BVd/TVd in the regenerated bone (PO2w and PO4w: P < 0.001 each). PO2w, 2 weeks postoperatively; PO4w, 4 weeks postoperatively.

Morphologic evaluation of micro-computed tomography (micro-CT) images of group W showed almost complete bone repair at 4 weeks postoperatively. Group P demonstrated reduced bone regeneration at 2 weeks; however, complete bone repair was achieved at 4 weeks postoperatively. Group P also showed an abundant periosteal reaction and endosteal bone formation. Groups C and PC exhibited reduced bone regeneration at 2 weeks postoperatively, similar to group P. Bone defects were bridged by new bone at 4 weeks postoperatively; however, the bridging tissues were thin and had persistent small holes (Fig. 2).

The quantitative BVd/TVd values at 2 weeks postoperatively were as follows: group W, 43.5 ± 3.4%; group P, 16.0 ± 1.0%; group C, 16.0 ± 0.7%; and group PC, 13.5 ± 1.5%. A two-factorial analysis of variance (ANOVA) for BVd/TVd showed that both, CIA and *P. gingivalis* administration had positive main effects and resulted in lower BVd/TVd (P < 0.001 for both). Multiple comparisons showed that BVd/TVd was the highest for group W (P < 0.001). The BVd/TVd values at 3 weeks postoperatively were as follows: group W: 73.0 ± 1.3%, group P: 64.0 ± 5.7%, group C: 43.7 ± 3.0%, and group PC: 41.0 ± 1.4%. Only CIA demonstrated a major effect (P < 0.001) and led to lower values. Groups C and PC demonstrated significantly lower values than group W (P < 0.001). At 4 weeks postoperatively, BVd/TVd were as follows: group W: 83.7 ± 2.6%, group P: 91.2 ± 1.2%, group C: 65.0 ± 3.3%, and group PC: 46.2 ± 2.4%. Only CIA demonstrated a major effect (p < 0.001) and led to lower values similar to those at 3 weeks postoperatively. Groups C and PC had significantly lower values than groups W and P, respectively. In addition, group PC had significantly lower values than group C (P < 0.001 for both) (Fig. 2 b–d). There was an interaction between CIA and *P. gingivalis* administration at 2 and 4 weeks postoperatively (P < 0.001, P = 0.25 and P < 0.001 at 2, 3 and 4 weeks post-operatively, respectively). Without *P. gingivalis* administration at 2 weeks postoperatively, simple effect analysis showed a higher level of BVd/TVd in mice without CIA [CIA(−)] than in those with CIA [CIA(+)]. Furthermore, CIA (−) mice not administered *P. gingivalis* (group W) had higher BVd/TVd values than those administered *P. gingivalis* (group P) (P < 0.001). The results at 4 weeks postoperatively showed that CIA (−) mice had higher BVd/TVd values than CIA (+) mice irrespective of *P. gingivalis* administration status. Furthermore, CIA (+) mice not administered *P. gingivalis* had higher values than those administered *P. gingivalis* (P < 0.001) (Fig. 2 e,f).

### Histological analyses of bone regeneration of cortical bone defects

**Figure 3:**
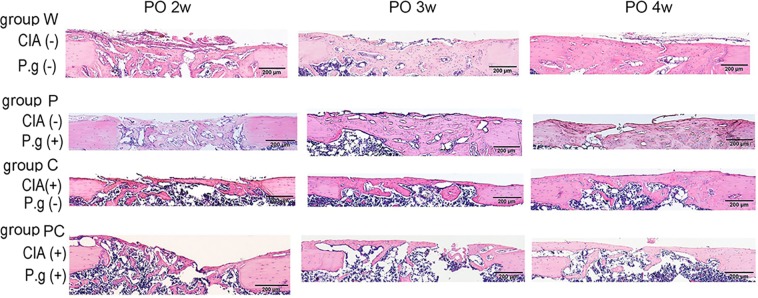
Representative images of 4-μm-thick sections from the midsagittal region of decalcified paraffin-embedded femurs were stained with haematoxylin and eosin. Groups C and PC showed insufficient bone regeneration at 4 weeks postoperatively (PO). n = 5–6 per group. *Black bar* represents 200 μm. CIA; collagen-induced arthritis, P.g; *Porphyromonas gingivalis* administration.

At 2 weeks postoperatively, haematoxylin and eosin (H&E) staining showed abundant new collagen in groups P, C, and PC, that were not recognised in mineralised bone using micro-CT. Groups W and P showed good bone regeneration at 4 weeks postoperatively; however, groups C and PC showed less bone regeneration. Defects in groups C and PC were almost bridged by the new bone superficially; however, the quantity of new bone was insufficient at the deep layer (Fig. 3). These findings at 4 weeks postoperatively were consistent with micro-CT findings.

### Bone resorption of the regenerated area was increased by CIA

**Figure 4:**
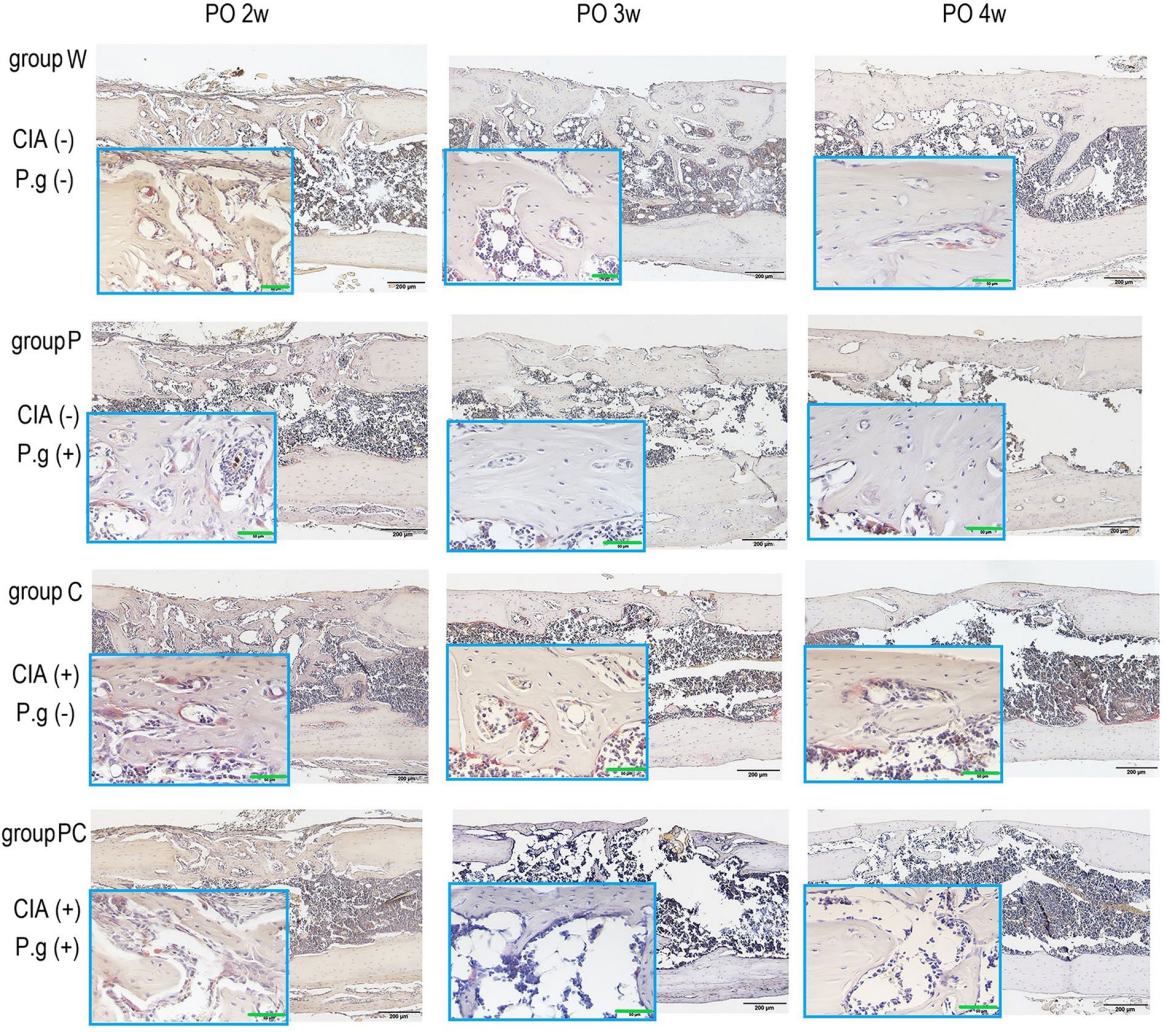
Midsagittal sections were made from decalcified paraffin-embedded femurs at 2 to 4 weeks postoperatively (PO) and stained for quantitative analyses of osteoclasts. Tartrate-resistant acid phosphatase-positive osteoclasts (red colour) adjacent to the newly formed collagen fibre (light beige) were higher in number in bones harvested from groups C and PC than those from groups W and P. n = 5–6 per group. *Black bar* represents 200 μm. *Green bar* represents 50 μm. CIA; collagen-induced arthritis, P.g; *Porphyromonas gingivalis* administration.

**Figure 5:**
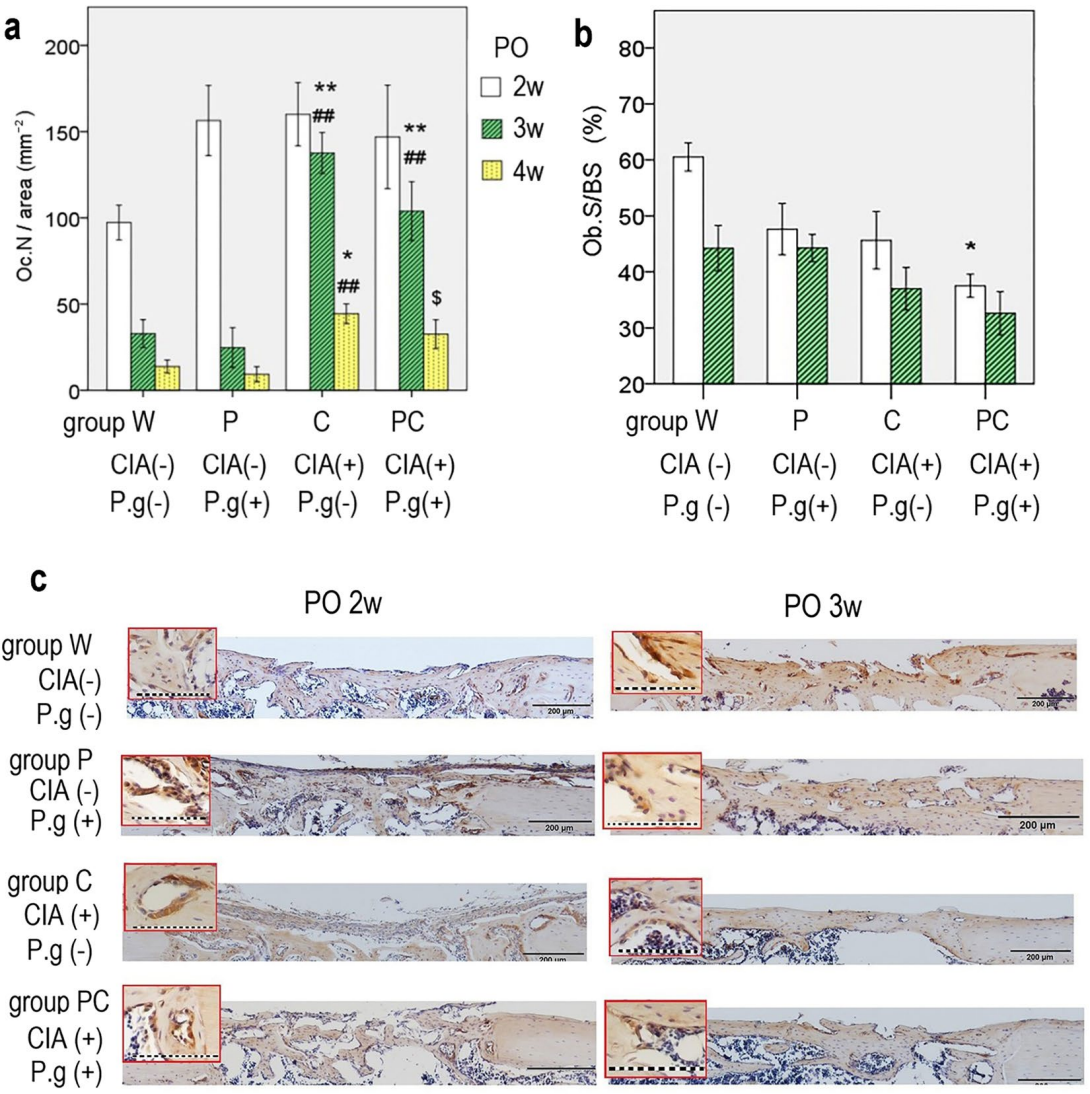
(**a**) Osteoclast number/decalcified bone area (Oc.N/area) at each time point are shown. Oc.N/area remained higher for groups C and PC at 3 and 4 weeks postoperatively. *P < 0.05, **P < 0.01 versus group W at the same time point. ^##^P < 0.01 versus group P at the same time point. ^$^P < 0.05 versus group C at the same time point. (**b**) Active osteoblast surface (Ob.S/BS) at 2 and 3 weeks postoperatively. Ob.S/BS of group PC is lower than that of group W at 2 weeks postoperatively (*p < 0.01). Data are presented as mean ± SEM. (**c**) Immunohistochemical staining of osteocalcin is shown. Group W showed more bone formation with the active osteoblast surface at 2 and 3 weeks postoperatively. Groups C and PC showed decreased bone formation and immunopositivity with osteocalcin. n = 5–6 per group. The magnification images are shown in red frame. *Black bar* represents 200 μm. *Dot line* represents 100 μm. CIA; collagen-induced arthritis, P.g; *Porphyromonas gingivalis* administration.

The osteoclast number/bone area (Oc. N/area) of groups C and PC with CIA was significantly increased compared with that of groups W and P, which did not have CIA at 3 and 4 weeks postoperatively (Figs. 4, 5a). *P. gingivalis* administration did not result in differences among the groups (2 weeks postoperatively: CIA and *P. gingivalis* administration, P = 0.19 and P = 0.29, respectively; 3 weeks postoperatively: CIA and *P. gingivalis* administration, P < 0.001 and P = 0.33, respectively; 4 weeks postoperatively: CIA and *P. gingivalis* administration, P = 0.019 and P = 0.11, respectively). There were no interactions between CIA and *P. gingivalis* administration at any time (Supplementary Information).

### Bone formation was decreased by CIA and *P. gingivalis* administration

The osteoblast surface/bone surface values at 2 weeks postoperatively were as follows: group W: 60.5 ± 2.3%, group P: 47.6 ± 4.2%, group C: 45.6 ± 4.7, and group PC: 37.5 ± 1.9%. Group PC had significantly lower values than group W (P = 0.002). The values at 3 weeks postoperatively were as follows: group W: 44.2 ± 3.7%, group P: 44.3 ± 2.0%, group C: 36.6 ± 3.2%, and group PC: 32.6 ± 3.5% (Fig. 5b,c). Both, CIA and *P. gingivalis* administration demonstrated the major effects of lower osteoblast surface/bone surface at 2 weeks postoperatively. However, only CIA demonstrated a major effect at 3 weeks postoperatively. No interaction was observed during these periods (Supplementary Information).

### Effects of CIA and *P. gingivalis* administration on bone mineral density

**Figure 6:**
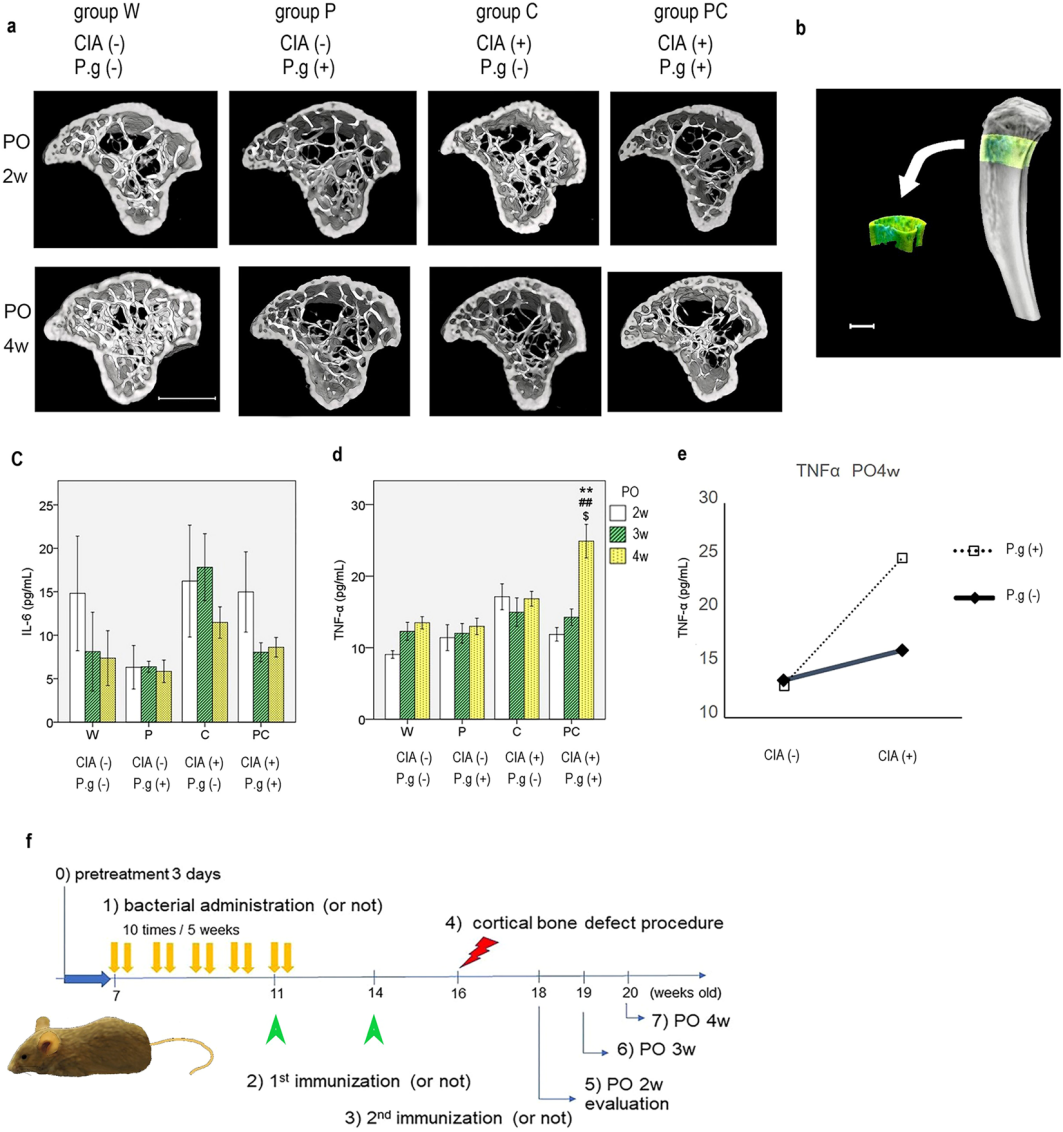
(**a**) Micro-CT images of the left proximal tibia in each group at 2 and 4 weeks postoperatively (PO) are shown. Groups P, C, and PC had decreased trabecular bone and increased cortical porosity at 4 weeks PO. n = 5–8/group. Axial slices of the proximal tibia view from the cranial side are shown. (**b**) The volume of interest is shown in the proximal tibia. Left, anterior tibia. *White bar* represents 900 μm. (**c**) Serum levels of interleukin-6 (IL-6), (**d**) tumour necrosis factor-α (TNF-α) in mice, and (**e**) the interaction at 4 weeks postoperatively (PO4w) are shown. Data are presented as mean ± SEM, n = 5–6 per group. **P < 0.01 versus group W at the same time point. ^##^P < 0.01 versus group P at the same time point. ^$^P < 0.05 versus group C at the same time point. CIA; collagen-induced arthritis, P.g; *Porphyromonas gingivalis* administration. (**f**) Schematic diagram of the experimental design. All mice were pretreated with drinking water including 0.1% kanamycin for 3 days (0). *Porphyromonas gingivalis* or phosphate-buffered saline was administered two times per week for 5 weeks (1). The first collagen sensitisation or normal saline injection was performed (2). Sensitised mice received a booster immunisation (3). The control group was injected with normal saline again. All mice underwent a cortical bone defect procedure in the bilateral femurs (4). Samples were obtained at 2, 3, and 4 weeks postoperatively (n = 5–8 per group) (5, 6, 7).

Micro-CT images showed differences in the bone microstructure, particularly trabecular microstructure, in the control and intervention groups. All intervention groups showed decreased trabecular bone and increased cortical porosity at 4 weeks postoperatively (9 weeks after the first sensitisation) (Fig. 6a,b). CIA and *P. gingivalis* administration affected a few micro-CT parameters and bone mineral density. CIA demonstrated a major effect on several parameters of osteoporosis at 2 weeks postoperatively (bone volume [BV]/tissue volume [TV], trabecular separation [Tb.Sp], trabecular spacing [Tb.Spac], tissue mineral density [TMD], and volumetric bone mineral density [vBMD]) and 4 weeks postoperatively (BV, BV/TV, trabecular thickness [Tb.Th], trabecular number [Tb.N], Tb.Sp, TMD, and vBMD). *P. gingivalis* administration did not have a major effect on osteoporosis at 2 weeks postoperatively; however, an effect was evident at 4 weeks postoperatively (in terms of BV, BV/TV, Tb.N, TMD, and vBMD). There were certain interactions with TMD (P = 0.04) and vBMD (P = 0.03) at 2 weeks postoperatively and with vBMD (P = 0.02) at 4 weeks postoperatively; however, there was no synergistic decrease (Supplementary Information).

At 4 weeks postoperatively, group W had a significantly increased vBMD compared with all other groups. Furthermore, group W had significantly increased BV/TV compared with groups C and PC. Moreover, the vBMD of group PC significantly decreased compared with that of group C (Tables 1, 2).

**Table 1:** Bone mineral density and bone microstructure of each group at postoperative 2 weeks.

parameter	(unit)	Group W; CIA(−) P.g(−)	Group P; CIA(−) P.g(+)	Group C; CIA(+) P.g(−)	Group PC; CIA(+) P.g(+)
TV	(mm^3^)	1.13 ± 0.07	1.09 ± 0.07	1.20 ± 0.04	1.24 ± 0.03
BV	(mm^3^)	0.14 ± 0.01	0.12 ± 0.01	0.11 ± 0.01	0.11 ± 0.01
BV/TV	(%)	12.8 ± 1.0	11.3 ± 0.94	8.98 ± 0.70	9.15 ± 0.57*
Tb.Th	(µm)	62.5 ± 4.36	55.0 ± 3.29	55.1 ± 1.84	51.1 ± 4.28
Tb.N	(1/mm)	1.29 ± 0.05	1.34 ± 0.07	1.15 ± 0.07	1.20 ± 0.08
Tb.Sp	(µm)	157 ± 7.59	156 ± 9.14	178 ± 6.49	179 ± 6.98
Tb.Spac	(µm)	220 ± 6.07	211 ± 7.65	233 ± 6.22	230 ± 8.54
TMD	(mg/cm^3^)	643 ± 32.6	524 ± 16.9*	500 ± 17.0**	506 ± 35.2*
vBMD	(mg/cm^3^)	80.9 ± 5.52	59.3 ± 4.97	44.7 ± 3.6**	41.5 ± 4.04**

Data are expressed as mean ± SEM, n = 6–8/group. CIA(+) or (−); with or without collagen-induced arthritis, P.g(+) or (−); with or without *P. gingivalis* administration, TV; tissue volume, BV; bone volume, Tb.Th; trabecular thickness, Tb.N; trabecular number, Tb.sp; trabecular separation, Tb.spac; trabecular spacing, TMD; tissue mineral density, vBMD; volumetric bone mineral density, *P < 0.05, **P < 0.01 compared with the group W.

**Table 2:** Bone mineral density and bone microstructure of each group at postoperative 4 weeks.

parameter	(unit)	group W; CIA(−) P.g(−)	group P; CIA(+) P.g(−)	group C; CIA(+) P.g(−)	group PC; CIA(+) P.g(+)
TV	(mm^3^)	1.13 ± 0.06	1.04 ± 0.02	1.13 ± 0.06	1.09 ± 0.05
BV	(mm^3^)	0.17 ± 0.02	0.11 ± 0.01	0.10 ± 0.01	0.07 ± 0.01*^,^^##^
BV/TV	(%)	14.5 ± 1.27	10.8 ± 0.66	8.16 ± 0.46*	6.40 ± 0.42**^,^^##^
Tb.Th	(µm)	58.9 ± 2.58	58.2 ± 1.81	48.5 ± 3.06	51.8 ± 3.44
Tb.N	(1/mm)	1.52 ± 0.10	1.13 ± 0.04*	1.17 ± 0.05	0.88 ± 0.06**^,^^#, $^
Tb.Sp	(µm)	162 ± 8.23	185 ± 9.96	190 ± 6.71	203 ± 11.3*
Tb.Spac	(µm)	220 ± 8.35	243 ± 9.46	238 ± 7.70	254 ± 12.0
TMD	(mg/cm^3^)	750 ± 49.0	592 ± 44.7	499 ± 19.2**	485 ± 18.6**
vBMD	(mg/cm^3^)	107 ± 9.18	64.7 ± 5.46*	40.6 ± 2.47**	30.7 ± 1.59**^,^^#, $^

Data are expressed as mean ± SEM, n = 6–8/group. CIA(+) or (−); with or without collagen-induced arthritis, P.g(+) or (−); with or without *P. gingivalis* administration, TV; tissue volume, BV; bone volume, BS; bone surface, Tb.Th; trabecular thickness, Tb.N; trabecular number, Tb.sp; trabecular separation, Tb.spac; trabecular spacing, TMD; tissue mineral density, vBMD; volumetric bone mineral density, *P < 0.05, **P < 0.01 compared with the group W, ^#^P < 0.05, ^##^P < 0.01 compared with the group P, ^$^P < 0.05 compared with the group C.

### Effects of CIA and *P. gingivalis* administration on the serum levels of inflammatory cytokines

Serum levels of IL-6 only exhibited significant differences at 3 weeks postoperatively (P = 0.27, P = 0.02, and P = 0.07 at 2, 3, and 4 weeks postoperatively, respectively). TNF-α levels of group PC at 4 weeks postoperatively were significantly higher than those of other groups. CIA and *P. gingivalis* administration showed an interaction at this time (P = 0.005), and the simple effect analysis showed higher TNF-α values for CIA with *P. gingivalis* administration (Fig. 6c–e, Supplementary Information).

## Discussion

The present study primarily indicates that CIA decreases bone regeneration in cortical defects of the femur, and the combination of CIA and *P. gingivalis* administration synergistically decreases bone regeneration during later phases (4 weeks postoperatively).

Although this study used an intramembranous ossification model, its findings are consistent with those of a previous report by Hu *et al*.^7^, who analysed endochondral ossification in CIA mice. They reported that CIA mice had lower callus volumes. In general, CIA mice experience high systemic inflammation. Similarly, Behrends^9^ reported reduced bone regeneration in femur cortical defects in a systemic inflammation model of mice with administration of lipopolysaccharide (LPS), a component of the bacterial cell wall in *P. gingivalis*.

Although single administration of *P. gingivalis* led to reduced bone regeneration compared to that in group W at 2 weeks postoperatively, active osteogenesis and periosteal reactions similar to those of group W were observed at 3 and 4 weeks postoperatively, indicating favourable bone restoration. Suppressed bone regeneration initially observed in the bone defects may have been compensated by active periosteal reactions of the peripherals. This mechanism may be explained by bone turnover facilitated through LPS-mediated osteoclastogenesis. We have previously showed that serum endotoxin (LPS) levels were significantly increased in *P. gingivalis* administered mice resulting in gut dysbiosis, dysfunction of gut barrier, and endotoxemia^33^. LPS facilitates osteoclastogenesis through the activation of the nuclear factor-kB/Toll-like receptors pathway^34^. Additionally, there was a difference between the volume of calcified bone on micro-CT and eosin-positive collagen fibres on histological imaging. Although configuring the general threshold of micro-CT at a high value equivalent to that of the cortical bone may have had an impact, Walsh *et al*. indicated that in a mouse serum transfer arthritis model, reductions in mineralisation of bone tissue (mineralising surface/bone surface) peripheral to the inflammatory articulation were less than that observed within the bone marrow ^35^; therefore, it is possible that similar changes may occur in bone regeneration. Histological imaging at 4 weeks postoperatively showed that in groups C and PC, the damaged bone was only restored on the surface, and not in the deep areas. This observation corresponded with the micro-CT results, and did not indicate an insufficiently mineralized condition; instead, it indicated a non-osteogenic condition.

Group P in the present study did not follow a pure periodontal disease model; it followed a dysbiosis model instead. Ciucci *et al*.^36^ and Ibáñez *et al*.^37^ used a dysbiosis model and showed that IL-17, a proinflammatory cytokine, directed inflammatory osteoclasts derived from dendritic cells towards the bone marrow. These cells, for which CX3CR1 is used as a marker, are different from osteoclasts induced by normal osteoclastogenesis. They induce TNF-generating CD4^+^ T cells and maintain and enhance inflammatory responses and bone destruction^37^. Although this study did not examine markers on the surface of lymphocytes, a previous study reported higher serum IL-17 levels in group PC than in group C, as well as changes in intestinal immunity accompanied by an increase in the T_H_17 ratio in the mesenteric lymph nodes^32^. Therefore, it is possible that osteoclasts similar to those reported by Ciucci *et al*. ^36^ may be induced. An increase in osteoclast counts in groups C and PC was observed in the present study. In addition, increased serum TNF-α levels were observed in group PC, suggesting the possibility that inflammatory osteoclasts were directed to the femoral bone marrow; this caused BVd/TVd reduction through excessive bone absorption in the bone restoration area.

Osteoblast surfaces are a parameter of bone regeneration (BVd/TVd). Osteoblast surfaces at 2 and 3 weeks postoperatively have an impact on late-stage bone repair; evaluation of found suppressed osteogenesis with both CIA and *P. gingivalis* administration at 2 weeks postoperatively. Although no synergistic suppression of osteogenesis was observed, significantly lower values were observed in group PC than in group W at this time. Therefore, it was considered that this led to reduced BVd/TVd levels at 4 weeks postoperatively. In terms of osteogenesis suppression functions in CIA, reports have shown increased expression of DKK-1, which is a Wnt signal antagonist, downstream of inflammatory cytokines, in association with osteoblast suppression^38,39^. Other reports have shown increased expression of B cells^40^. In terms of *P. gingivalis* administration, studies have reported that *P. gingivalis-*derived LPS increases sclerostin expression in osteocytes^41^. It is possible that these functions have an impact on the suppression of osteogenesis in the bone defect area.

In terms of the bone microstructure, decreased bone mass and mineral content were observed with CIA and *P. gingivalis* administration. These findings are also consistent with those of a previous report by Sandal *et al*. ^31^, who analysed experimental arthritis with *P. gingivalis* administration in mice. Although there was no synergistic reduction in bone mass and mineral content in group PC, the least amount of bone mineral content was observed. A large number of studies have shown that bone mass decreased with CIA and RA in a similar manner^39,42^. Increased inflammatory cytokines are believed to cause reductions in bone mass with CIA and RA; this is followed by the facilitation of osteoclast differentiation. Similar pathologies have been observed in dysbiosis; reports suggest that dysbiosis causes osteoclasts to induce reductions in bone mass; in a mouse model, probiotics have been administered to treat dysbiosis prevented bone mass reduction^43^. Moreover, a relationship has been suggested between changes in the intestinal microflora and osteoporosis in a few clinical cases^44^.

Serum analyses showed that TNF-α at 4 weeks postoperatively was significantly increased in group PC, which exhibited the most impaired bone healing. Anti-inflammatory cytokine antibody treatment has been reported to be effective for systemic inflammation models of diabetes^45,46^ and polytrauma^47^. Therefore, this intervention should studied further.

This study was the first to evaluate the synergistic effects of CIA and *P. gingivalis* administration on bone regeneration. At later stages of bone regeneration, there was a synergistic decrease in bone regeneration with CIA and *P. gingivalis* administration, and an increase in serum TNF-α. Additionally, the present study demonstrated that the combination of CIA and *P. gingivalis* administration resulted in the lowest bone mineral density.

This study had several limitations. We did not evaluate the cells or signalling pathways influencing bone regeneration sites in the dysbiotic intestinal environment. We only described intramembranous ossification, which is not suitable for making decisions regarding the healing of most clinical fractures.

In conclusion, our results suggest that interventions for dysbiosis or strong anti-inflammatory treatments for RA could improve fracture healing in RA patients. Further studies are needed to confirm our hypothesis.

## Methods

### Animals

Six-week-old male DBA/1 J mice were obtained from Japan SLC (Hamamatsu City, Japan). They were housed in small groups under specific pathogen-free conditions and were regularly fed chow and sterile water until the commencement of infection at 7 weeks of age. Before infection, mice were pre-treated with drinking water including 0.1% kanamycin^48^. They were randomly divided into the following four groups: saline as a negative control for collagen sensitisation plus vehicle as a control for bacterial administration (n = 24; group W), non-sensitisation with bacterial administration (n = 24; group P), collagen sensitisation without bacterial administration (n = 24; group C), and collagen sensitisation with bacterial administration (n = 24; group PC). Bacterial administration was performed for 5 weeks and collagen sensitisation twice for an additional 5 weeks. Two weeks after the second sensitisation to collagen (at age 16 weeks), all mice underwent a bilateral femoral uni-cortical defect procedure. Five to eight mice per group were euthanised at 2, 3, or 4 weeks postoperatively (Fig. 6f).

All animal experiments were conducted in accordance with the regulations and guidelines of scientific and ethical care and use of laboratory animals of the Science Council of Japan. All procedures used during this study were approved and performed in accordance with the guidelines of the Ethics Committee of the Institute of Biomedical Sciences of Niigata University (permit number: SA00167).

### Bacterial administration

We used *P. gingivalis* strain W83 in this study, which was maintained at the Research Unit for Oral-Systemic Connection of the Division of Oral Science for Health Promotion at Niigata University Graduate School of Medical and Dental Sciences. *P. gingivalis* was cultured in modified Gifu anaerobic medium (GAM) broth (Nissui, Tokyo, Japan) in an anaerobic jar (Mitsubishi Gas Chemical Co. Inc., Tokyo, Japan) in the presence of an AnaeroPack (Mitsubishi Gas Chemical Co. Inc.) for 48 hours at 37 °C. A total of 1 × 10^9^ colony-forming units of live *P. gingivalis* suspended in 100 µL of phosphate-buffered saline (PBS) with 2% carboxymethyl cellulose (Sigma-Aldrich, St. Louis, MO) was orally administered to each mouse through a feeding syringe twice per week for 5 weeks as described previously^32^. The control groups (W and C) underwent sham administration of 100 µL PBS with 2% carboxymethyl cellulose without bacteria. After administration, mice were allowed to eat and drink ad libitum.

### Arthritis induction and evaluation

After nine administrations of bacteria or vehicle, mice (11 weeks old) were immunised with bovine type II collagen (Chondrex, Inc., Redmond, WA) emulsified in complete Freund’s adjuvant (Chondrex) at a final concentration of 0.5 mg/mL of heat-killed *Mycobacterium tuberculosis H37 RA* (non-viable) and 0.1 mg/mL of collagen. Injections were administered subcutaneously in 100 µL volume of the emulsion at the base of the tail. At 3 weeks after primary immunisation (14 weeks old), mice were administered a booster immunisation dose with bovine type II collagen in incomplete Freund’s adjuvant (Chondrex). The control groups (W and P) were injected with 100 µL PBS twice instead of the emulsion.

Arthritis was scored by a blinded examiner using a visual assessment scoring (VAS) system with a scale of 0–4 per limb, as described previously^49^. Scoring was performed as follows: 0: no swelling or redness of paws or digits, 1: swelling and redness in one or two digits, 2: swelling and redness of the ankle or three or more digits or the midfoot, 3: swelling and redness of the ankle and midfoot or digits and midfoot, and 4: swelling and redness of the entire foot or ankylosis.

### Cortical bone defects in femurs

At 5 weeks after the primary immunisation (16 weeks old), mice were anesthetised with 0.1 mL of a mixture of medetomidine 0.2 mg/mL (Toronto Research Chemicals, Inc., Toronto, Canada), butorphanol tartrate 1.3 mg/mL (Wako Pure Chemical Industries, Ltd., Osaka, Japan), and midazolam 1 mg/mL (Sandoz Japan, Tokyo, Japan). Mice were placed in the supine position and the femurs were exposed using a medial approach to the muscles between the vastus medialis and rectus femoris, followed by splitting of the vastus intermedius. The periosteum was mechanically stripped using a scalpel before drilling. Cortical bone defects of the bilateral femurs were created using an electric drill (Kiso Power Tool Mfg. Co., Ltd., Osaka, Japan) with a 0.5-mm high-speed steel bur at 10,000 rpm. The operating field was frequently irrigated with normal saline to avoid thermal necrosis. Two longitudinally adjacent drill holes were created and connected. Oval holes 1.2 × 0.6 mm in diameter were subsequently created. Each hole was rinsed with saline using a 1-mL syringe and 31-gauge needle to wash away any bone debris in the cavity. After irrigation, the split muscle and skin incision were sutured in a layer-to-layer manner.

After recovery, animals were allowed to ambulate freely, and analgesics (butorphanol tartrate: 0.12 mg/mouse) were administered once per day for the first 48 hours. Analgesics were then given every other day to arthritic animals. Those that died during the postoperative period and those with fractures were excluded from further analyses.

### Micro-CT

The harvested left femurs were fixed in 4% paraformaldehyde solution and analysed using a micro-CT scanning device (Elescan; NSST, Tokyo, Japan) within 24 hours after euthanasia. The volume of interest (VOI) was manually set as the cylindrical region bordered by the edge of the primary cortical defect, with a long axis of 1200 μm and a thickness of 100 μm. This cylindrical region was defined as the three-dimensional (3D) TVd. The region was scanned using a fixed isotropic voxel of size 13.63 μm^3^ with a 1-mm aluminium filter using a voltage of 102 kVp and a current of 80 μA. Bone mineral density was determined using phantoms with a defined hydroxyapatite density (300, 400, 600, and 800 mg HA/cm^3^). Mineralised and poorly mineralised tissues were distinguished by a fixed global threshold of 700 mg HA/cm^3^ used for the new lamellar bone. The mineralised bone in the VOI was defined as the 3D BVd. The BVd/TVd (%) was used to evaluate bone regeneration in the cortical defect.

The harvested left tibias were fixed in 70% ethanol to measure volumetric bone mineral density and trabecular bone morphometry. The VOI of the tibia was the 1-mm-thick proximal tibial area starting from 0.2 mm below the lowest point of the growth plate. A fixed global threshold of 320 mg HA/cm^3^ was used for mineralised cancellous bone. The 3D microstructural imaging data were reconstructed, and the structural indices were calculated using TRI/3D Bon software (Ratoc System Engineering, Tokyo, Japan). Micro-CT parameters of bone mineral density and 3D bone morphometry were reported according to the international guidelines^50^.

### Histological analyses

To perform H&E and tartrate-resistant acid phosphatase (TRAP) staining, tissues fixed in 4% paraformaldehyde underwent decalcification in 10% ethylenediaminetetraacetic acid at 4 °C for 3 weeks. They were then embedded in paraffin after dehydration, and 4-µm-thick sections were created. Sectioning of the paraffin-embedded samples was performed along the sagittal plane.

H&E staining was performed using haematoxylin (Vector Laboratories, Inc., Burlingame, CA) for 3 minutes, followed by 10 seconds of eosin (Wako). TRAP staining was performed with Naphthol AS-BI phosphate (Sigma), sodium nitrite (Wako), 0.08 M L(+)-tartaric acid (Wako), and pararosaniline hydrochloride (Sigma) in 0.1 M of sodium acetate buffer (pH 5.0) at 37 °C in an incubator for 20 minutes, followed by nuclear counterstaining with haematoxylin. Under an Olympus light microscope (Olympus, Tokyo, Japan), TRAP-positive multinuclear giant cells adjacent to the collagen fibres were identified as osteoclasts. We counted the osteoclasts that were present in regions of bone regeneration and measured the area that consisted of new collagen (Olympus cellSens standard version 1.13; Olympus). The number of osteoclasts in the decalcified bone area (mm^−2^) was represented by the number of osteoclasts in the new collagen area.

### Immunohistochemical analyses

After antigen retrieval using 0.125% trypsin (Nichirei Bioscience, Tokyo, Japan) and quenching of endogenous peroxidase, the sections were incubated with rabbit polyclonal antibodies against osteocalcin (1:100 dilution; 20277-1-AP; Proteintech, Rosemont, IL, USA) for 90 minutes at room temperature. After washing, the sections were incubated for 30 minutes at room temperature with goat anti-rabbit immunoglobulin G secondary antibody (horseradish peroxidase) (1:500 dilution; 43R-1495; Fitzgerald Industries International, Inc., North Acton, MA). Tissue sections were visualised using 3,3′-diaminobenzidine (Simple Stain DAB; Nichirei Bioscience), followed by counterstaining with haematoxylin for 3 minutes. Images were acquired under the light microscope. We measured the perimeter of the new collagen (on the bone surface) in the area of bone regeneration and the length of the collagen surface with osteocalcin-positive osteoblasts (Olympus cellSens Standard; Olympus). The active osteoblast surface of the decalcified bone (%) was measured in relation to the decalcified bone surface.

### Serum analyses

Blood obtained from the mouse descending aorta was allowed to clot at room temperature. The blood clot was centrifuged for 15 minutes at 1000 × g. We collected supernatant serum and stored it at −20 °C until use. The IL-6 and TNF-α levels in serum were determined using a commercially available enzyme-linked immunosorbent assay (ELISA) (Thermo Fisher Scientific, Waltham, MA for IL-6; Proteintech for TNF-α) according to the manufacturers’ instructions. Absorbance at 450 nm was measured with an ELISA microplate reader (Bio-Rad Laboratories, Inc., Hercules, CA). The lower detection limits of IL-6 and TNF-α according to the ELISA kits were 0.2 pg/mL and 1.0 pg/mL, respectively.

### Statistical analyses

Results were analysed using Statistical Package for Social Science software version 21.0 (SPSS Inc, Chicago, IL) and presented as mean ± standard errors of the mean (SEM). Data were tested for normality using the Shapiro-Wilk test and for homogeneity of variance using Levene’s test. Considering the normal distribution and homogeneity of each group of data, we used the unpaired t test to analyse the arthritis scores of groups C and PC. To evaluate whether CIA and *P. gingivalis* administration affect bone regeneration, we used two-factorial ANOVA for most analyses; furthermore, we performed simple effect analyses using the Bonferroni correction for significant interactions and the Tukey’s test for comparisons between groups. We also used the Games-Howell test to perform multiple comparisons with heteroscedastic data. For the serum IL-6 analysis, we used the Kruskal-Wallis test as the data was non-normally distributed. The significance level was set at P < 0.05. The detailed results of the two-factorial ANOVA are shown in Supplementary data (Supplementary Information).

## Supplementary information

Detailed results (F value) of two-way analysis of variance

## Data Availability

The data sets generated and/or analysed during the present study are either included in the manuscript or available from the corresponding author on reasonable request.
