# Supplementary material for: Experimental arthritis and Porphyromonas gingivalis administration synergistically decrease bone regeneration in femoral cortical defects
Source: Sci Rep. 2019 Dec 27;9:20031. doi: 10.1038/s41598-019-56265-6 (PMC6934576; doi:10.1038/s41598-019-56265-6)
Supplement: Supplementary file 1 — Detailed results (F value) of two-way analysis of variance [file 41598_2019_56265_MOESM1_ESM.pdf]

Supplementary Information.

Experimental arthritis and *Porphyromonas gingivalis* administration synergistically decrease bone regeneration in femoral cortical defects

Go Okumura[1], Naoki Kondo[1], Keisuke Sato[2,3], Kazuhisa Yamazaki[3], Hayato Ohshima[4], Hiroyuki Kawashima[1], Akira Ogose[1], Naoto Endo[1]

[1]Division of Orthopedic Surgery, Department of Regenerative and Transplant Medicine, Niigata University Graduate School of Medical and Dental Sciences, Niigata, Japan

[2]Division of Periodontology, Department of Oral Science, Niigata University Graduate School of Medical and Dental Sciences, Niigata, Japan

[3]Research Unit for Oral-Systemic Connection, Division of Oral Science for Health Promotion, Niigata University Graduate School of Medical and Dental Sciences, Niigata, Japan

[4]Division of Anatomy and Cell Biology of the Hard Tissue, Department of Tissue Regeneration and Reconstruction, Niigata University Graduate School of Medical and Dental Sciences, Niigata, Japan

Correspondence to:

Go Okumura, MD, E-mail: goingok@hotmail.com

Supplementary Table. Detailed results (F value) of two-way analysis of variance

BVd / TVd of bone regenerated area.

parameter	CIA factor	P.g factor	CIA×P.g interaction
PO 2w	F(1,26)=59.9, P<0.001	F(1,26)=60.2, P<0.001	F(1,26)=41.6, P<0.001
PO 3w	F(1,23)=69.6, P<0.001	F(1,23)=2.60, P=0.12	F(1,23)=1.38, P=0.25.
PO 4w	F(1,25)=125.60, P<0.001	F(1,25)=3.96, P=0.06	F(1,25)=21.48, P<0.001

BVd/TVd; bone volume in cortical defect lesion / tissue volume in cortical defect lesion, CIA; collagen-induced arthritis, P.g; *Porphyromonas gingivalis* administration, PO; postoperative, P value with underline represents a significant difference.

Osteoclast number / decalcified bone regenerated area.

parameter	CIA factor	P.g factor	CIA×P.g interaction
PO 2w	F(1,25)=1.85, P=0.19	F(1,25)=1.19, P=0.29	F(1,25)=0.13, P=0.72
PO 3w	F(1,18)=51.2, P<0.001	F(1,18)=0.99, P=0.33	F(1,18)=0.99, P=0.33
PO 4w	F(1,21)=6.46, P=0.02	F(1,21)=2.78, P=0.11	F(1,21)=0.06, P=0.81

CIA; collagen-induced arthritis, P.g; *Porphyromonas gingivalis* administration, PO; postoperative, P value with underline represents a significant difference.

Osteoblast surface / decalcified bone surface.

parameter	CIA factor	P.g factor	CIA×P.g interaction
PO 2w	F(1,20)=10.7, P=0.004	F(1,20)=7.59, P=0.01	F(1,20)=0.34, P=0.54
PO 3w	F(1,20)=6.48, P=0.02	F(1,20)=0.34, P=0.57	F(1,20)=0.35, P=0.56

CIA; collagen-induced arthritis, P.g; *Porphyromonas gingivalis* administration, PO; postoperative, P value with underline represents a significant difference.

Bone mineral density in proximal tibia at postoperative 2 weeks.

parameter	CIA factor<br>F value(1,25), P value	P.g factor<br>F value(1,25), P value	CIA×P.g interaction<br>F value(1,25), P value
TV	F=3.61, P=0.07	F=0.008, P=0.93	F=0.55, P=0.47
BV	F=3.19, P=0.09	F=0.39, P=0.54	F=1.12, P=0.30
BV/TV	F=11.4, P=0.002	F=0.50, P=0.49	F=0.01, P=0.94
Tb. Th	F=2.23, P=0.15	F=0.23, P=0.14	F=0.21, P=0.65
Tb. N	F=4.04, P=0.06	F=0.62, P=0.44	F=0.001, P=0.97
Tb. Sp	F=7.39, P=0.01	F=0.001, P=0.98	F=0.02, P=0.88
Tb. Spac	F=4.59, P=0.04	F=0.61, P=0.44	F=0.15, P=0.70
TMD	F=8.08, P=0.009	F=3.98, P=0.06	F=4.90, P=0.04
vBMD	F=22.6, P<0.001	F=3.58, P=0.07	F=5.35, P=0.03

CIA; collagen-induced arthritis, P.g; *Porphyromonas gingivalis* administration, TV; tissue volume, BV; bone volume, Tb. Th; trabecular thickness, Tb.N; trabecular number, Tb.Sp; trabecular separation, Tb.Spac; trabecular spacing, TMD; tissue mineral density, vBMD; volumetric bone mineral density, P value with underline represents a significant difference.

Bone mineral density at postoperative 4 weeks.

parameter	CIA factor<br>F value(1,25), P value	P.g factor<br>F value(1,25), P value	CIA×P.g interaction<br>F value(1,25), P value
TV	F=0.19, P=0.67	F=1.41, P=0.25	F=0.17, P=0.68
BV	F=22.7, P<0.001	F=10.6, P=0.003	F=1.41, P=0.25
BV/TV	F=43.9, P<0.001	F=11.2, P=0.003	F=1.38, P=0.25
Tb. Th	F=7.03, P=0.01	F=0.17, P=0.69	F=0.41, P=0.53
Tb. N	F=17.4, P<0.001	F=23.2, P<0.001	F=0.52, P=0.48
Tb. Sp	F=5.15, P=0.03	F=3.28, P=0.08	F=0.29, P=0.61
Tb. Spac	F=1.87, P=0.18	F=3.44, P=0.08	F=0.09, P=0.77
TMD	F=25.4, P<0.001	F=6.24, P=0.02	F=3.49, P=0.07
vBMD	F=66.3, P<0.001	F=17.6 P<0.001	F=6.72, P=0.02

CIA; collagen-induced arthritis, P.g; *Porphyromonas gingivalis* administration, TV; tissue volume, BV; bone volume, Tb. Th; trabecular thickness, Tb.N; trabecular number, Tb.Sp; trabecular separation, Tb.Spac; trabecular spacing, TMD; tissue mineral density, vBMD; volumetric bone mineral density, P value with underline represents a significant difference.

Serum levels of TNF-α.

parameter	CIA factor	P.g factor	CIA×P.g interaction
PO2w	F(1,19)=8.18, P=0.010	F(1,19)=1.26, P=0.28	F(1,19)=7.65, P=0.012
PO3w	F(1,18)=2.33, P=0.14	F(1,18)=0.51, P=0.82	F(1,18)=0.00, P=0.97
PO4w	F(1,20)=25.7, P<0.001	F(1,20)=7.93, P=0.01	F(1,20)=10.0, P=0.005

TNF; tumor necrosis factor, CIA; collagen-induced arthritis, P.g; *Porphyromonas gingivalis* administration, PO; postoperative, P value with underline represents a significant difference.
